# Injury of the Head

**Published:** 1828-11

**Authors:** 


### INJURY OF THE HEAD.

### *Fracture of the Cranium. Abscesses discovered in the Liver and in the Lungs.*

James Parker, a robust young man, was admitted into St George's Hospital on the 2d of September. From the persons who brought him the following particulars were learnt: Whilst drunk in a pothouse on the 31st of August, he was beat with a stick about the body and head. He was taken home insensible, and bled to sixteen ounces in the course of the day, which produced no relief. From the time of the accident to that of his admission, he never regained the least sensibility.

He lay in an insensible, or rather semi-comatose condition, with a pulse slow and full; the pupils dilated; the surface neither blanched nor very cold; the breathing not stertorous, but deep drawn and heavy. Though not very restless when left to himself, he resisted all attempts to examine his condition with dogged and pertinaceous violence: he answered no questions, and seemed to comprehend none. Two inches, or more, above and behind the right ear was a pretty clean wound, confined to the scalp, and not having denuded the bone or pericranium. No fracture or depression was detected; the eye of that side was black with ecchymosis.

V.S. ad ℥viij.

He continued very restless through the night of the 2d, so much so indeed as to require the strait waistcoat. The bandage likewise slipped from the arm in the night, and occasioned the loss of eight ounces more blood.

On the morning of the 3d, there was little alteration from what had been observed on the preceding day. The treatment consisted in salines, with the sulphate of magnesia. He passed a bad night, endeavouring to get out of bed, &c.; and, between nine and ten A.M. of the 4th, was seized with convulsions. At one P.M. he had another fit. It began with a quivering of the lips, heaving of the chest, and convulsive affection of the muscles of the throat, and those which are engaged in the act of respiration: after a very short time, the breathing recommenced by snuffling through the nose, and gradually extended to the whole of the respiratory apparatus. The pupils before the attack were contracted, but afterwards dilated. The head was in the first instance drawn to the right, but subsequently fixed by the action of both the sterno-mastoidei. The portio dura of the right side was paralysed during the attack, the mouth being drawn to the left. The extremities, and especially the lower ones, were little affected. He had been bled to the extent of ℥xij. this morning.

It being evident, from the character of the symptoms, as well as their duration, that there existed something more than simple concussion, Mr. Keate, at two p.m., enlarged the wound, separated the pericranium, which was firmly attached to the bone, and discovered a fracture immediately anterior to the sutura lambdoidalis, unattended with perceptible depression. The trephine was "set on;" and the removal of the circular portion of bone disclosed a solid cushion of coagulum beneath. Two more pieces of bone were taken away by the trephine, and the margin of the clot, which was small, was apparently arrived at, though the fracture appeared to stretch downwards, perhaps to the basis. The first and the second pieces of bone that were removed shewed no bleeding whatever from the diploë; whilst the third, which was partly anterior to the coagulum, bled freely, and scarlet-coloured blood seemed to issue from the surface of the dura mater. The symptoms were not relieved; but no further indications remaining for trephining, the edges of the scalp, which had been crucially divided, were united by a suture in the form of a cross.

On the 7th, he had some return of sensibility, putting out his tongue when desired and motioned to do so, though perfectly unconscious of what was around him. He was not at all delirious, the breathing was easy, the pupils dilated, the surface of a natural warmth. Salines to be continued.

On the morning of the 8th, the sensibility was still more decided, and the symptoms altogether had a favorable cast. In the afternoon he was suddenly seized with a rigor, during which he was bled; and early on the 9th had another, which lasted very nearly an hour. At two p.m. he was quiet, and seemingly free from much pain. The pulse was more rapid than it had been for days, and, though not decidedly strong, was thrilling and jerky; tongue moist and white; surface hot; stools, as they had been all along, passed in bed. The sensibility was still on the increase, but the expression of the face was bewildered and anxious. The blood drawn last night much cupped and buffed; some which was taken this morning not so much so.

**Bleeding to be repeated in small quantities, its frequency being determined by the state of the blood and the pulse.**

11th.—The pulse is quick and full, surface hot, pupils dilated, and he complains of pain in the head, incessant and intense.

**V.S. ad ℥v. vel vi.**

Vesp.—Felt relieved by the bleeding, but the pulse is still frequent and hard. Blood much inflamed.

**Rep$^{r}$ V.S. ad ℥xij.—Rep$^{r}$ Mist.**

On the 12th, the pain of head was a little relieved, but the conjunctivæ and surface were assuming a yellow tinge. This bilious tint of skin was more marked on the 13th, and the symptoms were assuming the character of prostration and collapse. On the 14th, the surface was uniformly yellow, the mouth and teeth encrusted, the emaciation extreme. The sensibility was decidedly decreased, and, though evidently dying, he said he was "better." He died in the course of the 15th.

*Sectio cadaveris.*—The corpse was exceedingly emaciated, although on his admission the frame was robust and athletic.

Much extravasation was found beneath the scalp, particularly in the occipital region. On raising the skullcap, the dura mater was found to be perfectly sound at the part where the trephine had been applied. In the direction of the spine and transverse ridge of the occipital bone, where the fracture (as will be presently shown) had extended, a thin layer of blood was effused on the membrane, which was greatly inflamed, indeed actually sloughy. Opposite this inflamed portion of the dura mater, the tunica arachnoides was also inflamed, and covered with coagulable lymph. There was some, but not very much, fluid in the ventricles; no extravasation in, or rupture of, the brain; little or no effusion of blood at the basis.

The fracture extended from the spot where it first was discovered round the occipital bone, across the left branch of the lambdoid suture, obliquely over the petrous part of the left temporal bone, between the sella turcica and cuneiform process of the occipital bone; then over the right petrous portion, to the place from which we started, completing the circle, and literally breaking off the back of the head. Numerous and large depositions of lymph and pus existed in the liver, the texture of the viscus around being perceptibly engorged and inflamed. Similar appearances were discovered in the right lung; none in the left. The spleen and other viscera were healthy. The urine was bilious.

---

